# Exploring the effect of wiki-based writing instruction on writing skills and writing self-efficacy of Chinese English-as-a-foreign language learners

**DOI:** 10.3389/fpsyg.2022.1069832

**Published:** 2023-01-10

**Authors:** Jianchun Dai, Li Wang, Yongcheng He

**Affiliations:** ^1^School of Foreign Language Studies, Wenzhou Medical University, Wenzhou, Zhejiang, China; ^2^School of Foreign Studies, Wenzhou University, Wenzhou, Zhejiang, China

**Keywords:** web 2.0, IELTS, EFL writing, wiki-based instruction, writing skill, self-efficacy

## Abstract

As a popular Web 2.0 technology, wikis have gained momentum in educational contexts. To give further empirical support to the use of wikis in foreign language education, this research probed the usefulness of a wiki-based writing instruction on the writing skill and self-efficacy of Chinese English-as-a-foreign language (EFL) learners. For this purpose, 53 EFL students in a foreign language school in China were selected as the participants. The two classes were assigned to an experimental group (*N* = 25) and a control group (*N* = 28). Over a period of 3 months, the experimental group was instructed based on wiki-based writing instruction while the control group was taught traditionally. The data were collected by administering IELTS writing tasks and a writing self-efficacy scale employed for pre- and post-tests. The results of data analysis indicated that both classes substantially enhanced their writing skill and writing self-efficacy. Nevertheless, the experimental group performed better than the control group in terms of both dependent variables, leading the researchers to conclude that the wiki-based writing instruction was significantly effective in boosting writing skill and self-efficacy of Chinese EFL learners. These outcomes can offer some worthwhile implications for EFL instructors.

## 1. Introduction

Over the past few decades, there has been a widespread integration of internet and digital forms of communication in education and online application tools have become an integral part of teaching and learning (Santos et al., 2019; Huong and Hung, 2021; Liu et al., 2022). In the meantime, numerous Web 2.0 applications have been created which enable learners to generate, recreate, and access information more easily and effectively over the recent years. In the realm of second language (L2) education, Web 2.0 technological devices can allow learners to interact through L2 exposure and production, promote their attitudes as well as motivation, and use language to co-construct various L2 tasks (Krishnan et al., 2021; Rahimi and Fathi, 2022; Teo et al., 2022), particularly writing (Dizon, 2016; Kazazoglu and Bilir, 2021; Hung and Nguyen, 2022). As an example, Computer-Mediated Communication (CMC), namely emails, online discussion forums, and chats rooms may contribute to L2 learners’ meaningful engagement and communication, which in turn, might bring about fruitful language learning and teaching (Lin, 2015). In addition to these tools, more recent applications of the internet, such as blogs and wikis have gained momentum in the English-as-a-foreign language (EFL) contexts (Alharbi, 2015; Reinhardt, 2019), specifically for writing (Zhang et al., 2022).

Consistent with these developments and changes, various researchers have highlighted the significance of blogs or wikis for writing classes and have suggested wikis as a significant tool for collaborative work in the realm of education in general (Chu et al., 2019) and EFL context in particular (Su et al., 2019; Hosseini et al., 2021). Consequently, the adoption of wiki-based writing is coming to the fore in the context of L2 learning (Lai et al., 2016; Hosseini et al., 2020). Recognized as a Web 2.0 technology, wiki is a medium for collaborative writing which helps users to asynchronously compose a text online (Hsu, 2019; Khezrlou, 2022; Van Duong and Aslan, 2022). As Leuf and Cunningham (2001) maintained, wiki is a “freely expandable collection of interlinked web pages, a hypertext system for storing and modifying information – a database, where each page is easily edited by any user with a forms-capable Web browser client” (p. 14). Additionally, Wikis can also be utilized for writing assignments, specifically in blended or distance learning environments in different educational settings (Hudson, 2018). With the use of wikis, learners would be able to perform out-of-class collaborative writing tasks by reviewing and revising a text at any preferred time and place (Zou et al., 2016). Given the significance of wiki-based writing settings in L2 learning process (Su et al., 2019), it is of high importance to investigate this technology and its consequences in language learning and teaching. For instance, wiki-based writing contexts can significantly contribute to learners’ outcomes, such as boosting their L2 writing self-confidence (Rahimi and Fathi, 2021), writing proficiency (Li et al., 2014), and collaboration (Li and Zhu, 2017). However, despite the ample empirical evidence in other fields of study (e.g., Wichmann and Rummel, 2013; Lai et al., 2016; Stoddart et al., 2016; Pimlott and Tikasingh, 2021), there still exist a paucity regarding the investigation of wiki-based writing in the realm of Second Language Learning (SLA).

Due to the significance of dynamicity and context-specific nature of L2 learning, individual differences as well as the contextual factors have received more research attention by L2 scholars (Hiver and Al-Hoorie, 2016; Larsen-Freeman, 2016). As writing is a demanding productive skill which needs simultaneous processing of linguistic and affective factors, the role of individual differences becomes more boldfaced in this skill (Kellogg, 1996). According to Han and Hiver (2018), “psychosocial factors, such as learners’ writing specific emotions, self-efficacy and self-regulation, can regulate attention and cognitive engagement, and determine the level of effort learners will invest in the writing process” (p. 44). Consequently, given the critical role of individual and psychological factors in shaping learners’ writing (Han and Hiver, 2018), it seems warranted to investigate the potential effects of wiki-based instruction on L2 writing psychological constructs. Overall, in spite of the fact that a number of researchers have examined the effect of wikis on writing development (Li et al., 2014; Stoddart et al., 2016; Hsu, 2019; Rahimi and Fathi, 2021), this research area is still under-explored in EFL settings. More precisely, how wiki collaboration contributes to writing development is not very clear (Storch, 2011). Hence, in order to continue to pursue this line of inquiry to further inform our understanding of the nature and consequences of wiki-based writing instruction and to fill the identified lacuna, the current research examines the impact of wiki-based writing instruction on writing skills and writing self-efficacy of learners in the Chinese EFL context. As such, the two following research questions were formulated for the purpose of this study:

1. Does wiki-based writing instruction have any significant effect on the writing skills of Chinese EFL learners?
2. Does wiki-based writing instruction have any significant effect on the writing self-efficacy of Chinese EFL learners?

## 2. Literature review

### 2.1. Wiki-based writing

With the advent of Web 2.0 technologies, educational requirements have changed as well, specifically regarding writing process. Extending this into the realm of L2, computer-assisted language learning (CALL) and CMC have become critical tools by which technology can be implemented for the purpose of English writing (Xu and Yu, 2018; Hung et al., 2022). Among the various interactive technologies, wikis are innovative Web 2.0 tools which have been broadly adopted by EFL teachers in order to enhance the writing of their learners (Aydın and Yıldız, 2014; Alghasab et al., 2019). As a CMC open source (Chao and Lo, 2011), wiki is a platform by which users can purposely create and edit the content of web pages (Mak and Coniam, 2008). Wikis are distinguishable from traditional course systems in terms of their features such as open editing and social media tools, therefore, they are likely to improve and promote online collaboration of learners (Leung and Chu, 2009). According to Wang et al. (2011), wiki pertains to “active participation, connectivity, collaboration, and the sharing of knowledge and ideas among users” (p. 216). Despite the fact that it was mainly evolved for the purpose of documenting software patterns (Decker et al., 2007), wiki is currently being utilized in deferent educational settings to promote writing skills (Wichmann and Rummel, 2013; Pimlott and Tikasingh, 2021). Writing *via* wikis has recently become a focus of particular attention in various fields of study (i.e., EFL) and is now a significant part of many learner programs (Alharbi, 2020). As Wichmann and Rummel (2013) argued in their study, many learners are able to simultaneously collaborate as co-authors by remotely creating and editing the wiki-page in wiki-based writing process. Given the significance of cooperative learning in the context of L2 (Hung, 2019), Wiki platforms permits learners to write, edit and share the content collaboratively, and provides an environment for L2 teachers where they can examine and derive revision patterns from learners’ writing and set their writing strategies and instructions based on learners’ needs (Chin et al., 2015). Regarding Wikis’ user-friendly and learner-centered features, L2 teachers are provided with various opportunities to teach writing with process-oriented approaches (Ng and Lai, 2012). Wiki software has been commonly viewed as the most appropriate Web 2.0 tool to both promote online collaborative writing and pave the way for monitoring the co-authoring process by researchers (Wang, 2015; Jiang and Eslami, 2021; Teng, 2021; Cheung, 2022). According to Li (2011), wiki consists of three modules that are significantly conducive to collaborative writing: Edit allowing learners to write and revise writing texts on wiki pages, History showing all the changes made on the page, i.e., a specific color or deleted and inserted texts, Discussion providing the opportunity by which learners can negotiate writing tasks and meanings through asynchronous communication.

There exists a body of literature examining learners’ wiki writing process. For instance, in his study, Zorko (2007) concluded that wiki platforms can facilitate teacher-teacher and student-teacher collaboration, promote learners’ motivation, and develop learner autonomy. In another study in Spain, Vurdien (2020) explored how learners used wikis and smartphones to improve their writing skills. Collecting data from a sample of 21 EFL learners, the results indicated that the EFL learners held positive attitudes toward wiki writing and that wiki’s collaborative writing promoted the sharing of meaning and knowledge among participants. Gharehbagh et al. (2019) investigated the influences of written corrective feedback using wikis among 14 non-Malaysian ESL students in Kuala Lumpur. Utilizing the sample t-test, their results reported a major improvement regarding learners’ language factors such as content, language use, organization, and vocabulary. Additionally, it was indicated that wiki-based writing significantly motivated L2 learners in terms of learning English. Liou and Lee (2011) compared learners’ collaboratively and individually produced texts in wiki platform. To this end, 18 EFL students took part in this study. Their findings suggested that wiki-based collaborative writing can provide an environment in which learners are able to learn from each other. The results also revealed that EFL learners believed that collaborative activities can improve their writing performance. In another study in the United States, Elabdali (2016) examined the wiki-based collaborative writing in an L2 classroom. Gathering data from a number of 9 ESL learners, the authors reported that learners held the belief that creative writing tasks, particularly wikis contributed to their sense of agency and authenticity. Similarly, Hosseini et al. (2021) carried out a study on 72 EFL learners from a language institute in order to first, investigate the impact of wikis as a collaborative tool on learners’ writing fluency and second, to investigate their perceptions of using wikis. Administering a post-task attitude questionnaire entailing and four open-ended questions to the participants, the findings suggested that wiki tools significantly improved their writing fluency. Also, it was found that EFL students held positive attitudes toward the integration of wikis.

A review of the existing literature indicates that although the wiki-based writing has attracted much attention in various contexts like EFL (e.g., Chu et al., 2019; Ma, 2020; Vurdien, 2020; Rahimi and Fathi, 2021; Khezrlou, 2022), empirical evidence on its effects and consequences on L2 psychological factors and writing literacy is still fairly limited. Hence, as an attempt to bridge the identified gap in the literature, the present study sought to investigate the contributions of wiki-based writing instruction to EFL learners’ psychological and writing resources such as writing competency and self-efficacy. In another words, we investigated the effect of learners’ wiki-based writing instruction on their writing skills and writing self-efficacy in the EFL context.

### 2.2. Self-efficacy

As Bandura (1986) noted, self-efficacy is commonly viewed as one’s belief or judgment regarding his/her own capabilities in order to perform a particular task or action. The higher an individual’s level of self-efficacy, the more that individual is confident that he/she is able to complete a particular action at a specific level. Self-efficacy is rooted in Bandura’s (1977) social cognitive theory and Rotter’s (1966) locus of control theory, and is a multidimensional and domain-specific construct (Sezgintürk and Sungur, 2020). It is argued that self-efficacy is the basis of individuals’ motivation, in fact, without a person’s judgment of his own ability to effectively complete a task; it is not easy to motivate people to undertake it (Sezgintürk and Sungur, 2020). Extending this into education, self-efficacy pertains to learners’ belief about their own capacities and capabilities to achieve a particular educational objective or outcome, (e.g., learners’ capabilities to do well on a test or get desired grades in school Olivier et al., 2019). Zimmerman (2000) maintained that learners’ self-efficacy can play a predictive role in affecting their motivation to learn, their performance, effort, persistence, and emotional reactions to setbacks during learning. Furthermore, Bandura (1989) noted that there is a strong and positive relationship between student’s self-efficacy and the amount of effort they exert, which in turn, can lead to their higher academic achievements regardless of actual capability. In their study, Huang and Mayer (2019) concluded that learners’ self-efficacy exerted a strong influence on their achievement in an online course. In fact, it is apparent that self-efficacy and students’ academic achievement and desired outcomes are positively and significantly correlated (Hayat et al., 2020; Tus, 2020).

Since self-efficacy is context-dependent (Bandura, 1986), online self-efficacy can be viewed as a warranted and worthwhile construct which is concerned with learners’ beliefs in their competence in employing technology, online learning, and sustaining social interaction in online educational contexts (Tezer et al., 2018; Uzunboylu et al., 2020). As Stephen et al. (2020) suggested, self-efficacy plays a pivotal role in the development of learners’ online persistence by which they are able to focus on their nontraditional, online needs, skills, and characteristics.

### 2.3. Writing self-efficacy

As mentioned earlier, self-efficacy is a domain-specific construct (Bandura, 2006), meaning that self-efficacy measures are in essence specific within one area of knowledge but not others. As Pajares and Valiante (2006) indicated, measures of self-efficacy must include “an understanding of both the domain under investigation and its different features, as well as of the types of capabilities the domain requires and the range of situations in which these capabilities might be applied” (p. 162). Therefore, as one can argue, this view highlights the value of framing writing self-efficacy in a way that can reflect learners’ confidence regarding effectively meeting their psychological, linguistic, and behavioral challenges during the writing process (Bruning et al., 2013; Yilmaz Soylu et al., 2017). First coined by Pajares (2003), writing self-efficacy can effectively control a writer’s thoughts, feelings, and actions (Hetthong and Teo, 2013). As a context specific construct (Chea and Shumow, 2017), writing self-efficacy is referred to as learners’ beliefs about their abilities and capacities to effectively write a text (Schunk and Swartz, 1993; Woodrow, 2011; Sun et al., 2021). As affected by both learners’ physiological and emotional reactions to an activity and their past experience and verbal feedback from peers (Bandura, 1986), writing self-efficacy can be conducive to learners’ desired writing performance (Golparvar and Khafi, 2021). As Bruning et al. (2013) suggested, writing self-efficacy consists of three dimensions: Ideation, as the first step of the writing task, refers to learner’s capability to create ideas, Convention referring to learner’s capability to convey the created ideas *via* linguistics skills, and Self-regulation pertaining to learners’ self-management and control of their own thoughts, feelings, and actions and also to learners’ perception about their cognitive and linguistic capacities while writing a text. EFL learners’ confidence in English writing can not only reflect their attitudes toward writing but it also can indicate learners’ likelihood to master or avoid the writing process (Zhang and Guo, 2012). As Hetthong and Teo (2013) suggested in their study, if EFL learners are confident enough to complete a writing task, regardless of the fact that whether they are competent or not, they are more inclined to invest effort in coping with setbacks and difficulties while writing a text. Moreover, a growing number of studies have highlighted the fact that EFL students who believed in their abilities to use their capacities and regulate their actions while writing, in return were more likely to invest effort in the writing process and seize the writing opportunities (e.g., Kim et al., 2015). For instance, Sun and Wang (2020) carried out a study to investigate the association between writing self-efficacy and writing self-regulated learning strategies and writing proficiency of EFL learners. To do so, two different questionnaires were administered to a sample of 319 EFL students in the context of China. Employing confirmatory factor analysis, their results demonstrated that both writing self-efficacy and writing self-regulated learning strategies positively and significantly predicted EFL learners’ writing proficiency. In another study in China, Woodrow (2011) explored the association between learners’ writing self-efficacy and writing performance. Collecting data from a sample of 738 EFL learners, the findings indicated that there was a strong and positive relationship between writing self-efficacy and writing performance of learners. In addition, it was revealed that writing self-efficacy negatively related with learners’ anxiety.

It is worth noting that few if any studies have focused solely on the relationship between writing self-efficacy and wiki-based instruction, however, writing self-efficacy is argued to be promoted by other online tools and platforms. For example, Alberth (2019) investigated the influence of a social media (i.e., Facebook) on learners’ writing performance and writing self-efficacy among 64 EFL students. The results demonstrated that Facebook not only improved the EFL learners’ writing performance but also promoted their writing self-efficacy. In a study, Rahimi and Fathi (2021) conducted a sequential explanatory mixed-methods study to examine the influence of wiki-mediated collaborative writing on EFL learners’ writing performance, writing self-regulation, and writing self-efficacy. To this end, 67 Iranian EFL students took part in this study. The results of the quantitative data revealed that both wiki-mediated and non-wiki collaborative writing instructions promoted EFL learners’ writing performance, writing self-regulation, and writing self-efficacy. Additionally, in terms of the qualitative data, it was indicated that writing mediations positively predicted EFL learners’ writing proficiency in the wiki platform. In the similar vein, Fathi et al. (2019) examined the impacts of a blog-mediated writing course on learners’ writing motivation, self-efficacy, and self-regulation. To this aim, the authors collected data from a sample of 46 Iranian EFL students. Employing an explanatory sequential design, their results indicated that Blog-Mediated Writing Instruction significantly contributed to writing motivation and writing self-regulation of EFL learners. Nevertheless, it was found that the blog-mediated writing course decreased EFL students’ writing self-efficacy.

### 2.4. Wiki-based writing instruction and writing skills

A mounting number of studies have delved into the effectiveness of wiki-based writing on writing skills of learners (e.g., Chao and Lo, 2011; Sun and Qiu, 2014; Hsu, 2019; Vurdien, 2020; Rahimi and Fathi, 2021; Khezrlou, 2022). For instance, Li et al. (2014) examined the impacts of wiki-based collaborative writing on writing ability and writing perceptions of learners. Their findings demonstrated that wikis positively affected learners’ writing ability. In another study, Pae (2007) explored the association between wiki-based English writing classes and learners’ writing proficiency and anxiety among 15 EFL students. The findings revealed that the wiki-based instruction had a positive effect on learners’ writing proficiency but had insignificant impact on their writing anxiety. In Brazil, Franco (2008) probed the effect of wiki-based tools on learners’ writing skills. Administering a survey to sample of 18 EFL learners, the results reported a predictive role of wikis in developing learners’ writing skills. In another study, in Iran, Akbari and Erfani (2018) investigated the impacts of wikis on writing skill of EFL learners. The results of statistical analysis indicated that utilizing wikis was associated with higher levels of writing skills among participants. In the same vein, Kioumarsi et al. (2018) investigated the influences of wikis and wiki-based process writing instruction on EFL learners’ writing abilities. To do so, 16 Iranian EFL students took part in this research. The findings indicated that wiki-based instruction significantly contributed to EFL learners’ writing skills and promoted their motivation and autonomy in writing.

The aforementioned literature highlights the critical role that wiki tools play in the process of learners’ writing skills. Although there is a growing body of literature that deals with wikis in the L2 context, there has been a scarcity of research on its association with learners’ writing proficiency and skills in the context of EFL. In addition, no other study, so far, has attended simultaneously to the associations between wiki tools and EFL learners’ writing self-efficacy and writing skills. Hence, as an attempt to fill that void, the present study delves into the effects of wiki-based instruction on learners’ writing self-efficacy and writing skills in the context of EFL.

## 3. Materials and methods

### 3.1. Participants

This was a quasi-experimental study in which two intact groups (53 students in total) were employed as the participants. In total, 53 Chinese EFL students took part in this study. These participants were students of two classes from a foreign language school in Zhejiang Province of China. Given the criteria of availability and willingness to participate, convenience sampling was used to select these EFL students who included male and female students. The two classes were assigned to an experimental group (*N* = 25) and a control group (*N* = 28). Participants’ age ranged from 18 to 23 (*M* = 22.36, SD = 3.27) and they reported that they had the experience of learning English from 7 to 15 years (*M* = 9.02, SD = 2.89). This language school was preparing EFL students to take IELTS and whose purpose was to elevate the language competencies of the students in the four skills. In this course, the students had enrolled in the writing skill course with duration of 3 months, 2 h per week. The two groups were taught by a trained IELTS teacher who was also familiar with wikis.

### 3.2. Instruments

#### 3.2.1. English proficiency test

In order to investigate whether the EFL students were not substantially different in terms of global English proficiency, Oxford Placement Test (OPT) was administered to two groups. This test is considered as an appropriate test of proficiency which can gage English abilities of various students with differing levels (Allan, 2004). To compare the two groups in terms of English proficiency, their average scores were compared *via* performing an independent samples *t*-test. The result revealed that there no substantial difference existed between them, verifying their homogeneity before starting the intervention. The Cronbach’s alpha reliability of the test was 0.87 in this research.

#### 3.2.2. Writing performance test

EFL learners’ writing skill was assessed using sample IELTS tasks selected from ‘Collins writing for IELTS’ (Williams, 2011). To this end, two sample tasks were administered as pre- and post-tests. The content validity of the two sample tasks was checked carefully by two domain experts specializing in L2 writing research in terms of content and suitability. In other words, the experts evaluated how well the tasks could measure the writing skill of the participants. As for scoring the written tasks, IELTS academic writing rubric (University of Cambridge ESOL Examinations, 2011) was used. The written tasks were rated by another writing expert who had the experience of using IELTS writing rubric. The inter-rater reliability coefficient as measured by Cohen’s Kappa turned out to be 0.84.

#### 3.2.3. Writing self-efficacy scale

Participants’ self-efficacy in doing writing tasks was measured using a 9-item self-report scale developed by Han and Hiver (2018). This scale aims to assess the respondents’ perceived confidence in doing written English tasks particularly in terms of organization, content, grammar as well as format, vocabulary, and sentence structure. The domain experts were again consulted to judge the content validity of this scale by checking the items and they agreed that this self-report scale could be used for measuring writing self-efficacy of the participants. This scale was administered twice as pre- and post-test. The reliability coefficients of this scale, as estimated by Cronbach’s Alpha formula, were 0.82 and 0.85 in the pre- and post-tests, respectively.

### 3.3. Operationalization of the independent variable

Wiki-based instruction was use for doing the EFL collaborative writing tasks in the experimental group. Initially, the instructor gave the learners a general discussion on how to employ wiki for doing writing tasks collaboratively. He explained the features of wiki including achieving, revising, and commenting in the forum. The participants of the experimental group were divided in groups of three or four individuals in order to construct their own wikis for doing writing tasks. These students were requested to do writing tasks together outside the class by giving feedback to each other on various aspects of writing in terms of language, content, and organization *via* wiki collaborative discussions. They were also asked to do a written task (e.g., to write about a topic) each session. There was a warm-up stage for each task prior to using wiki pages in order to activate the background knowledge of the participants. The instructor ensured that participants have sufficiently participated in group discussions and collaborative writing by monitoring wiki discussions and activities. In addition, tried to encourage the less active participants to become more actively involved in the wiki activities. The teacher also aided the students in providing more constructive and peer mediations on different aspects of writing.

On the contrary, the participants of the control group were requested to do similar writing tasks collaboratively but without the use of wikis or any other technology devices. In fact, all the procedures, content, and tasks of the two groups were the same except for the fact that the collaborative writing tasks were not wiki-mediated. These students were also required to give peer-feedback to each other with regard to different aspects of writing organization, language use, and content. There was a schema activation phase like that of the experimental group in which the students were involved in brainstorming about the topic. Like the wiki group, the students of the control group were also divided in groups of three or four. The teacher also checked the group activities and peer mediations of the participants.

### 3.4. Data collection

In the first session of the course (week 1), the instructor provided the students with a general discussion of the purpose of the course. Then the pre-tests including the sample IELTS task 1 and writing self-efficacy scale was administered to the students of both groups. At the end of the treatment which lasted for about 13 weeks, the post-tests were given to the participants. More specifically, the last session (week 13) was devoted to students’ completing the writing self-efficacy scale and doing the sample IELTS task 2.

### 3.5. Data analysis

The data analysis was carried out using IBM SPSS program (version 23). To address the research questions of this research, both descriptive and inferential statistics were utilized. With regard to the descriptive statistics, mean, standard deviation, skewness, kurtosis, and reliability coefficients were calculated. Concerning the inferential statistics, one-way analysis of covariance (ANCOVA) was used for each research question. In each ANCOVA analysis, the pre-test scores were a covariate, the post-test scores were considered as the dependent variables, and the instruction type with two levels (i.e., wiki-based or conventional) was the independent variable.

## 4. Results

Before calculating the descriptive statistics and performing ANCOVAs, some pre-requisite assumptions were checked. With regard to checking the normality of the data, skewedness and kurtosis values were taken into account. More specifically, the ratios of skewedness and kurtosis statistics to the standard errors were referred to.

As seen in Table 1, the ratios lay within the range from −1.96 to +1.96, confirming that the data were normally distributed. As required by ANCOVA analyzes, some other preliminary checks were made to ensure that the assumptions were all met and not violated. More particularly, the other assumptions of linearity, homogeneity of variances, homogeneity of regression slopes, and reliable estimation of the covariate were all investigated. These investigations revealed that none of the assumptions were violated, confirming that the ANCOVAs could be run.

**Table 1:** Testing the normality of the data.

	*N*	Skewedness (Std. Error)	Kurtosis (Std. Error)
Writing 1	53	0.327 (0.293)	−0.337 (0.622)
Writing 2	53	−0.304 (0.301)	−1.21 (0.634)
Self-efficacy 1	53	−0.439 (0.303)	−0.118 (0.627)
Self-efficacy 2	53	−0.412 (0.299)	−0.375 (0.616)

As for the first research question which was concerned with exploring the impact of wiki-based writing course on EFL writing skill, as Table 2 displays, the writing mean score of the wiki group increased from 55.84 (SD = 8.99) to 88.92 (SD = 10.87) on the post-test. In the same vein, the mean score of writing skill for the control group rose from 56.53 (SD = 9.42) to 66.67 (SD = 9.97) on the post-test. Nevertheless, after adjusting for the covariate (writing pre-test scores), a significant difference was observed between the groups in terms of writing skill, *F* (1, 50) = 59.51, (*p* < 0.001, partial eta squared = 0.54; see Table 3). This outcome shows that the students of the wiki group enhanced their writing skill substantially greater than the those in the control group, verifying that the use of wikis in the writing instruction substantially enhanced the writing skill of the participants.

**Table 2:** Descriptive statistics for pre- and post-tests scores.

	Group	*N*	Mean	Std. Deviation	Std. Error mean
Pre writing	Experimental	25	55.84	8.99	1.79
	Control	28	56.53	9.42	1.78
Post writing	Experimental	25	88.92	10.87	2.17
	Control	28	66.67	9.97	1.88
Pre efficacy	Experimental	25	20.46	4.65	0.93
	Control	28	20.1	4.28	0.62
Post efficacy	Experimental	25	30.38	7.45	1.49
	Control	28	22.8036	4.9	0.73

**Table 3:** The results of ANCOVA on writing skill.

Source	Type III sum of squares	df	Mean square	*F*	Sig.	Partial eta squared
Covariate (pre-test)	74.987	1	74.987	0.689	0.41	0.014
Between-subjects	6470.38	1	6470.38	59.416	0	0.543
Within-subjects	5444.96	50	108.899			

Research question 2 was set to explore the effect of wiki-based instruction on the writing self-efficacy of Chinese EFL learners, As seen in Table 2, the descriptive statistics data illustrate that the mean score of the experimental group on the writing self-efficacy score was 20.46 (SD = 4.65) in the pre-test and this value was raised to 30.38 (SD = 7.45) on the post-test. Likewise, the mean score of writing self-efficacy for the control group was raised from 20.10 (SD = 4.28) on the pre-test to 22.80 (SD = 4.90) on the post-test. After adjusting the covariate (i.e., self-efficacy scores), ANCOVA results (see Table 4) showed that a significant difference existed between the two groups in terms of writing self-efficacy, *F* (1, 50) = 23.11, (*p* < 0.001, partial eta squared = 0.31). This result highlighted the fact that the use of wikis in the writing instruction significantly contributed to improving the writing self-efficacy of Chinese EFL students.

**Table 4:** The results of ANCOVA on writing self-efficacy.

Source	Type III sum of squares	Df	Mean square	*F*	Sig.	Partial eta squared
Covariate (pre-test)	179.401	1	179.401	5.73	0.02	0.103
Between-subjects	723.829	1	723.829	23.119	0	0.316
Within-subjects	1565.409	50	31.308			

## 5. Discussion

The current research aimed to investigate the effect of wiki-based writing instruction on the writing skills and writing self-efficacy of Chinese EFL learners. Regarding the purposes of the current research and the formulated research questions, the results of the ANCOVAs by comparing the two dependent variables (i.e., writing skills and writing self-efficacy) between two study groups on the posttest scores after controlling for the pretest scores yielded two notable findings.

First, the investigation of research question one revealed that the use of wiki-based writing improved writing performance of EFL learners. This result is in accordance with previous research on the usefulness of wikis in affecting learners’ writing skills (e.g., Li et al., 2014; Akbari and Erfani, 2018; Khezrlou, 2022; Van Duong and Aslan, 2022). In addition, this finding supports Hsu’s (2019) study which demonstrated that wiki collaborative Pae writing positively improved learners’ writing performance. As one can argue, wiki-mediated collaborative writing can significantly promote writing skills of EFL learners. Following what Ma (2020) demonstrated in his study, learners using online wiki platforms are more likely to effectively peer correct and give feedback on the writing activities and contribute to their writing performance. Therefore, one possible explanation or this finding might be in light of the fact that due to the learner-centered feature of the wiki space, EFL learners might be provided with the opportunity to conveniently and collaboratively monitor and enhance their peers’ writing proficiency. These results are in agreement with the empirical research of Liou and Lee (2011) who reported that wiki-based collaborative writing tasks can provide convenient environments in which students can learn from each other, which in turn, may lead to their enhanced writing. Furthermore, the findings can be justified in the light of the study of Zorko (2007) who points out that wiki-based instruction may contribute to learners’ autonomy as they can be in control of their own learning and construct their own knowledge independently, which eventually, might promote their writing skills.

Second, the results of the data analysis for examining the second research question indicated that the use of wiki-based writing improved writing self-efficacy of Chinese EFL learners. The outcomes of this study are in line with the study by Rahimi and Fathi (2021) who suggested that wikis can significantly enhance the writing self-efficacy of EFL learners. The results partially corroborate the ideas of Alberth (2019) who reported that online application tools had positive effects on facilitating learners’ writing self-efficacy. In addition, the findings are partially in line with those of Xu et al. (2011) who demonstrated that online environments, in which learners have no limitations of time and space, may boost students’ writing self-efficacy. A possible explanation seems valid in this regard: since learners use wiki tools for their writing tasks, they can collaborate and share ideas with other learners using wikis and find solutions for problems or be resistance in the face of difficulties while writing a text, and since solving problems and being resistance when facing setbacks is correlated with higher self-efficacy (Garza et al., 2014); therefore, it can be conjectured that wiki tools can contribute to learners’ self-efficacy. Furthermore, following Bandura (1977), given the fact that students can write collaboratively in a comfortably and friendly online environment without any specific limitations which may promote their writing self-efficacy, we postulate that wiki-based instructions can increase learners’ positive attitudes and perceptions regarding their writing skills and performance. Another rationale behind this prediction might be that while using wikis, learners can receive positive feedbacks from peers which in turn can enhance writing self-efficacy as feedbacks are positively associated with learners’ self-efficacy (Wang and Wu, 2008). As such, EFL learners receiving positive feedback from other peers while writing can experience higher levels of writing self-efficacy (Rahimi and Fathi, 2021). However, this finding is partially in contrast with those of Fathi et al. (2019) who noted that blog-mediated writing course decreased learners’ self-efficacy.

## 6. Conclusions and implications

To lend further support to the importance of online application tools and Web 2.0 in EFL contexts, this study sought to investigate the effect of wiki-based instruction on the writing skills and writing self-efficacy of Chinese EFL learners. As demonstrated by the results, the current study indicated that wiki was an effective Web 2.0 tool for promoting and improving writing skills and writing self-efficacy of the EFL learners. The current research was the first attempt to probe the simultaneous relationships among the constructs of wiki-based instruction, writing skills, and writing self-efficacy of EFL students. Given the fact that wiki tools have a significant role in affecting writing self-efficacy and writing performance, EFL teachers and methodologists should consider wiki-based instruction intervention programs in order to facilitate EFL learners’ integration of wiki tools. The outcomes of the study can also offer some implications to policy makers and EFL teacher educators so that they can create more appropriate conditions for EFL instructors to design an effective online wiki context for EFL learners. Training teachers to use wikis more effectively will help their students to carry out writing collaboratively and successfully.

In the same vein, further initiatives should be taken to enhance EFL learners’ writing self-efficacy and writing abilities by paying particular attention to utilizing effective strategies and approaches to promote their beliefs of competence in writing tasks and their abilities to practically write texts. Despite the fact that the results generated valuable insights into the benefits of implementing wiki tools in EFL learning context, the findings complete just a small piece of a complex puzzle and therefore further research is needed to contribute to a better understanding and some ambiguity about the nature, features, dimensionality, and consequences of integration of Web 2.0 tools, specifically wikis in the realm of EFL learning and teaching. In addition, it is apparent that self-efficacy plays an integral role in L2 learning (Goetze and Driver, 2022), since it can significantly contribute to different skills of EFL students, particularly their writing (Bai and Guo, 2018). Hence, L2 officials and policy makers should make an attempt to bolster the self-efficacy of EFL learners in classrooms for a fruitful English learning.

Concerning the limitations of this research, it is worth noting that we utilized just a quantitative research method. Therefore, next researchers are invited to triangulate their findings with adding qualitative or mixed-methods research designs to shed more light on the utility of wikis in EFL writing courses. Also, the external validity of the results will be enhanced if the future researchers replicate studies by recruiting larger samples of participants with various proficiency levels in EFL contexts other than China. Given the potential effectiveness of wikis, future researchers are encouraged to explore the effects of wikis on other EFL skills and components.

## Data availability statement

The original contributions presented in the study are included in the article/supplementary material, further inquiries can be directed to the corresponding author.

## Ethics statement

The studies involving human participants were reviewed and approved by Wenzhou Medical University Academic Ethics Committee. The patients/participants provided their written informed consent to participate in this study.

## Author contributions

All authors listed have made a substantial, direct, and intellectual contribution to the work and approved it for publication.

## Conflict of interest

The authors declare that the research was conducted in the absence of any commercial or financial relationships that could be construed as a potential conflict of interest.

## Publisher’s note

All claims expressed in this article are solely those of the authors and do not necessarily represent those of their affiliated organizations, or those of the publisher, the editors and the reviewers. Any product that may be evaluated in this article, or claim that may be made by its manufacturer, is not guaranteed or endorsed by the publisher.
